# Cortical thickness analysis in temporal lobe epilepsy using fully Bayesian spectral method in magnetic resonance imaging

**DOI:** 10.1186/s12880-022-00949-5

**Published:** 2022-12-21

**Authors:** Iman Sarbisheh, Leili Tapak, Alireza Fallahi, Javad Fardmal, Majid Sadeghifar, MohammadReza Nazemzadeh, Jafar Mehvari Habibabadi

**Affiliations:** 1 Department of Biostatistics, School of Public Health, Hamadan University of Medical Sciences, Hamadan, Iran; 2 Department of Biostatistics, School of Public Health and Modeling of Noncommunicable Diseases Research Center, Hamadan University of Medical Sciences, Hamadan, Iran; 3 Research Center for Molecular and Cellular Imaging, Advanced Medical Technologies and Instruments Institute (AMTII), Tehran University of Medical Sciences, Tehran, Iran; 4 Department of Statistics, Faculty of Science, Bu-Ali Sina University, Hamadan, Iran; 5 Physics and Biomedical Engineering Department, Tehran University of Medical Sciences, Tehran, Iran; 6 Department of Medicine, Isfahan University of Medical Sciences, Isfahan, Iran; 7 Biomedical Engineering Department, Hamedan University of Technology, Hamedan, Iran

**Keywords:** Cortical thickness, Markov chain Monte Carlo, Matérn correlation, Spatial dependence, Temporal lobe epilepsy

## Abstract

**Background:**

Temporal lobe epilepsy (TLE) is the most common type of epilepsy associated with changes in the cerebral cortex throughout the brain. Magnetic resonance imaging (MRI) is widely used for detecting such anomalies; nevertheless, it produces spatially correlated data that cannot be considered by the usual statistical models. This study aimed to compare cortical thicknesses between patients with TLE and healthy controls by considering the spatial dependencies across different regions of the cerebral cortex in MRI.

**Methods:**

In this study, T1-weighted MRI was performed on 20 healthy controls and 33 TLE patients. Nineteen patients had a left TLE and 14 had a right TLE. Cortical thickness was measured for all individuals in 68 regions of the cerebral cortex based on images. Fully Bayesian spectral method was utilized to compare the cortical thickness of different brain regions between groups. Neural networks model was used to classify the patients using the identified regions.

**Results:**

For the left TLE patients, cortical thinning was observed in bilateral caudal anterior cingulate, lateral orbitofrontal (ipsilateral), the bilateral rostral anterior cingulate, frontal pole and temporal pole (ipsilateral), caudal middle frontal and rostral middle frontal (contralateral side). For the right TLE patients, cortical thinning was only observed in the entorhinal area (ipsilateral). The AUCs of the neural networks for classification of left and right TLE patients versus healthy controls were 0.939 and 1.000, respectively.

**Conclusion:**

Alteration of cortical gray matter thickness was evidenced as common effect of epileptogenicity, as manifested by the patients in this study using the fully Bayesian spectral method by taking into account the complex structure of the data.

## Introduction

Epilepsy is considered as a common chronic neurological disorder affecting individuals of various ages, sexes, races, social classes, and geographical locations [1]. According to World Health Organization (WHO), about 50 million people have epilepsy globally; eighty percent of them live in low- and middle-income countries [2]. Epilepsy affects brain areas and generates episodes of seizure, putting a heavy neurobiological, cognitive, psychological, and social burden on the patients and their families [1]. The most common type of epilepsy in adults is Temporal Lobe Epilepsy (TLE) which is the typical type of drug-resistant epilepsy that requires surgical treatment [3, 4]. Accurate localization of seizure onset affects the success of the surgery, and due to the lack of a precise abnormality localization, surgery is not a solution for about 30% of TLE patients [5].

TLE is mainly characterized by hippocampal atrophy [6]. The seizures in TLE start from the temporal lobe and cause structural and functional abnormalities in the brain [7]. There have been conducted several studies on the gray and white matters using magnetic resonance imaging (MRI), including volumetry, voxel-based morphometry, and cortical thickness measurement [8, 9]. They found structural changes “extended to temporolimbic and frontocentral regions” in the gray matter and morphological and microstructural abnormalities in the white matter within/beyond the temporal lobes [6, 10]. Brain atrophy deterioration due to the seizure-induced damages over time has also been reported by studies [11]. These changes can affect the seizure-related surgery results and cognitive impairments across various domains [6]. Therefore, determining abnormal regions/areas of the brain in TLE is very important [12].

Atrophy is a progressive condition in different regions of the cortex in patients with drug-resistant epilepsy [11], and widespread patterns of neocortical atrophy in people with epilepsy have been confirmed by studies [13, 14]. It has been well-established that structural/functional changes in a TLE patient’s brain may correlate with alterations in cognitive function in a number of domains. Additionally, many patients with TLE are evaluated to epilepsy surgery, as they continue to have seizures despite optimal medical management. Therefore, it is crucial to lateralize the seizure focus [15–17]. In this regard, studies assessed the structural asymmetry in patients with TLE in the diffusion properties of brain white matter and the subcortical gray matter tracts [17]. Moreover, some attempts have been made to compare the atrophy in patients with left and right TLE.

Keller et al. (2002) investigated gray matter abnormalities in patients with TLE [18]. They observed a reduced preferential gray matter concentration in the anterior hippocampus in patients with left hippocampal atrophy and posterior hippocampus in patients with the right hippocampal atrophy. They also observed a gray matter concentration reduction in the right dorsal prefrontal cortex in both left and right hippocampal atrophy patients. Pail et al. conducted a study and determined changes in gray matter volume in patients with TLE [19] and Jaber et al. (2021) reported widespread structural brain abnormalities and cortical thinning in patients with TLE [20]. These studies have examined cortical thickness patterns in patients with TLE. However, only a few have considered the potential spatial dependencies between different regions of the cerebral.

Brain regions are functionally correlated, and also evidenced to be spatially structured, reflecting the structural architecture and internally homogenous areas of the cortical regions of the human brain [21–26]. Accounting for these spatial dependencies in the cortical thicknesses obtained from MRI plays a crucial role in getting efficient and valid inference [27]. This is also the case for the cortical thickness in patients with TLE due to the spatial patterns of regional atrophy. Studies have confirmed better discrimination between MRI scans related to normal and impaired individuals by accounting for spatial patterns instead of a univariate analysis of each region like a vertex-by-vertex regression (28). Integrating spatial information of the regions of interest into the cortical thickness information prevents the effect of noise and outliers on measurements. Spatial data was also previously investigated by researches using other methods like independent component analysis and self-organizing map for brain functional network analysis [29, 30].

There have been suggested several approaches for taking into account the spatial correlation between regions in analyzing MRI data, including low-rank models [31], Spatio-Spectral mixed effects model for functional magnetic resonance imaging (fMRI) time courses [32, 33], Spatio-Temporal model with spatially varying coefficients and multi-resolution error structure [34] which all suffer from computational expensiveness issue. Among others, Reich et al. (2018) introduced a new fully Bayesian spectral approach for imaging data analysis that takes into account the spatial dependencies throughout brain imaging data and applied the method to investigate associations between cortical thickness and Alzheimer’s disease [35]. There are several advantages of using this model. A Bayesian approach which is considered in their model accounts for uncertainty in all parameters of the model [33, 36]. The model also allows for the effect of the covariates to vary spatially and shrinks it spatially to encourage sparseness. The functional connectivity between distant regions of the brain is captured by the residual covariance as “a combination of stationary covariance for flexible local dependence and a low-rank nonstationary covariance.” The large covariance matrix is handled through a projection into the spectral domain to de-correlate the outcome [35]. They conducted several simulation studies indicating that their model provided efficient estimates for the association parameters and a better inference compared to the settings that do not consider spatial dependencies. Their proposed model is efficient for MRI data as the number of voxels, or anatomic regions is usually greater than the number of subjects (p > n). However, to the best of our knowledge, no study has utilized the Bayesian spectral approach to analyze the MRI data related to epilepsy patients to compare their cortical thickness between left and right TLE and healthy people. The MRI data obtained from the epilepsy patients are also correlated, and previous studies used the typical independent samples t-test or a vertex-by-vertex regression to compare the cortical thickness of patients and healthy controls region-by-region (a univariate analysis). This leads to an increase in the size of the type I error. This study aimed to utilize a fully Bayesian spectral approach based on Markov Chain Monte Carlo sampling methods to characterize the patterns of cortical atrophy and to perform cortical thickness analysis in TLE to investigate cortical thickness abnormalities in the TLE cerebral cortex. We considered a node-based cortical thickness analysis.

## Methods

### Subjects

In this study, we used a data set consisting of information on refractory TLE patients, including 19 patients with left TLE and 14 patients with right TLE. The participants were enrolled among the patients referred to the epilepsy long-term monitoring (LTM) clinics. Also, a number of 20 healthy controls with no history of neurological/mental disorders were enrolled (see Table 1 for demographic characteristics of participants). A team consisting of neurologists, epileptologists, neuropsychologists, and a neurosurgeon collaborated to determine the side of laterality in a multi-disciplinary pre-surgical decision-making session by using several criteria, including (1) descriptions/manifestation of seizure semiology; (2) ictal Electroencephalographic (EEG); (3) ictal epileptogenic zone; (4) interictal–irritative zone; and (5) MRI findings. Exclusion criteria consisted of (1) having disabling cognitive impairments; (2) having other neurological diseases; (3) having any serious systemic/psychological diseases; (4) aged > 55 and < 16 years; (5) having substance/alcohol abuse history; (6) being pregnant; and (7) Breastfeeding. “For establishing MRI-proven mesial temporal sclerosis, approximated or fully recognizable abnormal alterations in hippocampal imaging attributes including shrinkage in volume and shape on T1-weighted images or hyper signal intensity on T2 FLAIR (fluid attenuated inversion recovery) images were examined. Along with MRI evidence, to establish the epileptogenic side, diagnostic procedures have been performed based on seizure semiology and video-EEG monitoring compatible with TLE” [20].

**Table 1:** Clinical and investigative characteristics of patients

Group	Number of cases	Gender (M/F)	Age mean $\pm$ SD
HC	20	10/10	27.95 ± 6.32
Left TLE	19	11/8	32.10 ± 8.47
Right TLE	14	10/4	28.28 ± 6.28
ALL	53	31/22	29.53 ± 7.29

*HC* Healthy controls, *TLE* Temporal lobe epilepsy

### Image acquisition

MRI data were acquired at the Iranian National Brain Mapping Laboratory (NBML) utilizing a 64-channel phased-array head coil on a 3-Tesla scanner (Siemens Prisma, Erlangen, Germany) using software version "Syngo MR E11" (NMBL). For clinical diagnosis, anatomic pictures were acquired using a standardized MPRAGE IR procedure for transverse T1-weighted images with the following imaging parameters: TR = 1840 ms, TI = 900 ms, TE = 3.4 ms, flip angle = 8°, matrix = $224 \times 224$, inplane resolution = $1.0 \times 1.0$ mm. The thickness of the two slices is 1.0 mm, and the pixel bandwidth is 250 Hz/pixel.

### Cortical thickness measurement

The cortical thickness measurement was done using Free-Surfer software through the following steps: (1) Skull stripping; (2) inflation of the folded surface tessellation patterns; (3) intensity normalization; (4) segmentation; and (5) tessellation of the gray/white matter border as well as automatic correction. Then, the white and gray matter, as well as the gray/cerebrospinal fluid surfaces, were obtained by using a deformable surface algorithm so that the representations of the cortical thicknesses throughout the brain were obtained based on the intensities and information produced in deformation procedures. The representations were determined as the shortest distance between the gray/cerebrospinal fluid border and the gray/white border at each vertex on the tessellated surface. Thickness measurements were then projected into the inflated surface of each subject's brain reconstruction.

The Desikan parcellation atlas [37] was used to analyze all cortical thickness regions between the control and patient groups using the fully Bayesian spectral method. The mean cortical thickness of each parcellated region was measured in Free-Surfer using the Query Design Estimate Contrast (QDEC) tool.

## Statistical analysis

A brief review of the fully Bayesian spectral method for spatial data was provided below; for a complete review we refer the readers to Reich et al. (2018) [35].

### Model description in the spatial domain

Let ${\text{Y}}_{{\text{i}}} \left( {\text{v}} \right)$ be the response (here, cortical thickness) at spatial location ${\text{v}} = \left( {{\text{v}}_{1} ,{\text{v}}_{2} ,{\text{v}}_{3} } \right)^{{\text{T}}}$ for subject $i$. The model for subject $i$ is

1 $$\begin{aligned} & {\text{Y}}_{{\text{i}}} \left( {\text{v}} \right) = \mathop \sum \limits_{{{\text{k}} = 0}}^{{\text{p}}} {\text{X}}_{{{\text{ik}}}} {\text{B}}_{{\text{K}}} \left( {\text{v}} \right) + \mathop \sum \limits_{{{\text{j}} = 1}}^{{\text{J}}} {\text{Z}}_{{\text{j}}} \left( {\text{v}} \right)\upgamma _{{{\text{ij}}}} + {\text{E}}_{{\text{i}}} \left( {\text{v}} \right) \\ & k = 0,1, \ldots ,p \\ & i = 1,2, \ldots ,m \\ & v = 1,2, \ldots ,n \\ \end{aligned}$$

where the ${\text{B}}_{{\text{K}}}$ is the spatially varying fixed-effect process,${\text{Z}}_{{\text{j}}}$ is a set well-known basis functions that explains the spatial structure at a large scale, $\upgamma _{{\text{i}}} = \left( {\upgamma _{{{\text{i}}1}} ,\upgamma _{{{\text{i}}2}} , \ldots ,\upgamma _{{{\text{iJ}}}} } \right)^{{\text{T}}}$ is the vector of corresponding random effects; and ${\text{E}}_{{\text{i}}} \left( {\text{v}} \right)$ is a small-scale spatial deviation. The residual spatial process ${\text{E}}_{{\text{i}}}$ is a mean-zero Gaussian process with an isotropic Matérn covariance function [38] as follows:

2 $${\text{Cov}}\left[ {{\text{E}}_{{\text{i}}} \left( {{\rm v}} \right),{\text{E}}_{{\text{i}}} \left( {{{\rm v}}^{\prime } } \right)} \right] =\upsigma ^{2} {\text{I}}\left( {{{\rm v}} = {{\rm v}}^{\prime } } \right) +\uptau ^{2} {\text{M}}_{{{\rm v}}} \left( {\frac{{{{\rm v}} - {{\rm v}}^{\prime } }}{{\upvarphi }} } \right)$$

where ${\text{M}}_{{\upnu }}$ is the 3D-Matérn correlation ${\text{ M}}_{\upnu } \left( {\text{h}} \right) = \frac{{2^{{1 -\upnu }} }}{{\Gamma \left(\upnu \right)}}\left( {3{\text{h}}\sqrt {\upvarphi } } \right)^{{\upnu { }}} {\text{R}}_{\upnu } \left( {3{\text{h}}\sqrt {\upvarphi } } \right),$ and ${\text{R}}$ is the modified Bessel function of the second kind.

### Model description in the spectral domain

To de-correlate the Matérn process and to analyze the entire spatial domain, the data were transformed to the spectral domain. Because the data are defined on a sphere, the spherical harmonics transformation (SHT) was utilized for the $B_{k}$ and $E_{i}$. The $E_{i}$ ($B_{k}$ is expanded similarly) is represented as follows:

3 $${\text{E}}_{{\text{i}}} \left( {\text{v}} \right) = \mathop \sum \limits_{{{\text{l}} = 0}}^{{\text{L}}} \mathop \sum \limits_{{{\text{m}} = - {\text{l}}}}^{{\text{l}}} \frac{1}{{\sqrt {2{\text{l}} + 1} }} {\text{S}}_{{\text{l}}}^{{\text{m}}} \left( {{\text{s}}_{1} ,{\text{s}}_{2} } \right)\upxi _{{\text{i}}} \left(\upomega \right)$$

where ${\text{S}}_{{\text{m}}}^{{\text{l}}} \left( {{\text{s}}_{1} ,{\text{s}}_{2} } \right)$ stands for the spherical harmonic functions, $\upomega = \left( {\text{l}} \right.,\left. {\text{m}} \right)$ and $\upxi _{{\text{i}}} \left(\upomega \right)$ indicate the unknown coefficients.

The spherical harmonic functions are as follows:

4 $${\text{S}}_{{\text{l}}}^{{\text{m}}} \left( {{\text{s}}_{1} ,{\text{s}}_{2} } \right) = {\text{P}}_{{\text{l}}}^{{\text{m}}} \left[ {\cos \left( {{\text{s}}_{1} } \right)} \right] {\text{e}}^{{ - {\text{ims}}_{2} }}$$

where ${\text{P}}_{{\text{l}}} \;{\text{stands}}\;{\text{for}}$ the associated Legendre polynomial. A full representation of the process requires ${\text{L}} = \infty$; nevertheless, here the process was approximated with a finite L. Since the SHT is a linear operator, the spatial model in Eq. (1) becomes as follows:

5 $$\widetilde{{{\rm Y}}}_{{\text{i}}} \left(\upomega \right) = \mathop \sum \limits_{{{\text{k}} = 1}}^{{\text{p}}} {\text{X}}_{{{\text{ik}}}} {\tilde{{\rm B}}}_{{\text{K}}} \left(\upomega \right) + \mathop \sum \limits_{{{\text{j}} = 1}}^{{\text{J}}} {\tilde{{\rm Z}}}_{{\text{j}}} \left(\upomega \right)\upgamma _{{{\text{ij}}}} + {\tilde{\text{E}}}_{{\text{i}}} \left(\upomega \right)$$

Let us denote the unique real values extracted from the complex data in the spectral domain as $\widetilde{{\text{Y}}}_{{\text{i}}} \left(\upomega \right)$, ${\tilde{\text{E}}}_{{\text{i}}} \left(\upomega \right)$, ${\tilde{\text{Z}}}_{{\text{j}}} \left(\upomega \right)$ and ${\tilde{\text{B}}}_{{\text{K}}} \left(\upomega \right)$ for the processes ${\text{Y}}_{{\text{i}}} \left( {\upnu } \right),{\text{E}}_{{\text{i}}} \left( {\upnu } \right),{\text{Z}}_{{\text{j}}} \left( {\upnu } \right)$ and ${\text{B}}_{{\text{k}}} \left( {\upnu } \right)$, respectively, for frequency ω. The spectral process ${\tilde{\text{Z}}}_{{\text{j}}} \left(\upomega \right)$ will be nonzero for all ${\text{j}}$ and $\upomega$. Therefore, to improve computational efficiency, we set ${\tilde{\text{Z}}}_{{\text{j}}} \left(\upomega \right) = 0$ for terms with $\upomega \notin { }{\mathcal{L}}$ and select ${\mathcal{L}}$ to include roughly ${\text{J}}$ terms.

### Priors and computing details

The model presented in “Statistical analysis” section and 4 can be summarized as follows. For terms with $\upomega \epsilon {\ominus } {\mathcal{L}}$

$${\tilde{\text{Y}}}_{{\text{i}}} \left(\upomega \right)\mathop \sim \limits^{{\text{ indep }}} {\text{Normal}} \left( {\mathop \sum \limits_{{{\text{k}} = 1}}^{{\text{p}}} {\text{X}}_{{{\text{ik}}}} {\tilde{\text{B}}}_{{\text{K}}} \left(\upomega \right) + \mathop \sum \limits_{{{\text{j}} = 1}}^{{\text{J}}} {\tilde{\text{Z}}}_{{\text{j}}} \left(\upomega \right)\upgamma _{{{\text{ij}}}} ,{\tilde{\lambda }}\left( {\upomega |\uptheta } \right)} \right)$$

$$\upgamma _{{\text{i}}} \mathop \sim \limits^{{\text{ indep }}} {\text{Normal}}\left( {0 ,\Sigma } \right)$$

$${\tilde{\text{B}}}_{{\text{k}}} \left(\upomega \right)\mathop \sim \limits^{{\text{ indep }}} {\text{Normal}}\left( {\mathop \sum \limits_{{{\text{j}} = 1}}^{{\text{J}}} {\tilde{\text{Z}}}_{{\text{j}}} \left(\upomega \right) \upbeta _{{{\text{kj}} }} ,{\tilde{\lambda }}\left( {\upomega |\uptheta _{{\text{k}}} } \right)} \right)\quad {\text{k}} = 0,1, \ldots ,{\text{p}}$$

$$\upbeta _{{0{\text{j}}}} \mathop \sim \limits^{{\text{ indep }}} {\text{Normal}}\left( {0 ,\updelta _{0}^{2} } \right)$$

$$\upbeta _{{{\text{kj}}}} \mathop \sim \limits^{{\text{ indep }}} {\text{Normal}}\left( {0 ,\updelta _{{{\text{kj}}}}^{2} } \right) \quad\updelta _{{{\text{kj}} }} \sim{\text{halfcauchy}}\left( {\updelta _{{\text{k}}} } \right)\quad {\text{k}} > 0$$

For terms with $\upomega \notin { }{\mathcal{L}}$

$$\widetilde{{\text{Y}}}_{{\text{i}}} \left(\upomega \right) \mathop \sim \limits^{{\text{ indep }}} {\text{Normal}}\left( {\mathop \sum \limits_{{{\text{k}} = 1}}^{{\text{p}}} {\text{X}}_{{{\text{ik}}}} {\tilde{\text{B}}}_{{\text{K}}} \left(\upomega \right),{\tilde{\lambda }}\left( {\upomega |\uptheta } \right)} \right)$$

$${\tilde{\text{B}}}_{{\text{k}}} \left(\upomega \right)\mathop \sim \limits^{{\text{ indep }}} {\text{Normal}}\left( {0,{\tilde{\lambda }}\left( {\upomega {|}\uptheta _{{\text{k}}} } \right)} \right)\quad {\text{k}} = 1,2, \ldots ,{ }P$$

$\widetilde{{\text{Y}}}_{{\text{i}}} \left(\upomega \right)$ and ${\tilde{\text{B}}}_{{\text{l}}} \left(\upomega \right)$ are independent across $\upomega$ after the spectral transformation. The Matérn parameters are re-parameterized from $\uptheta = \left( {\upsigma ^{2} ,\uptau ^{2} ,\phi ,{\upnu }} \right)$ (and in the same way for the $\theta_{k}$) to overall variance $\upnu =\upsigma ^{2} +\uptau ^{2}$, the logit of the spatial variance proportion ${\text{r}} = {\text{logit}}\left[ {\frac{{\uptau ^{2} }}{{\upsigma ^{2} +\uptau ^{2} }}} \right]$, and the log range and smoothness, $\upnu ^{\prime } = \log \left(\upnu \right)$ and ${\upvarphi }^{^{\prime}} = {\text{log}}\left( {\upvarphi } \right)$. The following uninformative priors are considered to complete the Bayesian model.

$$\sum \sim{\text{Inv}}Wishart\left( {{\text{J}} +\upvarepsilon } \right.,\left. {\frac{{{\upnu }_{3} }}{{{\text{J}} +\upvarepsilon }}{\text{I}}_{{\text{J}}} } \right)$$

$${\upnu },{\upnu }_{0} ,{\upnu }_{1} ,\updelta _{0} ,\updelta _{{\text{k}}} \sim{\text{InvGamma}}\left( {\upvarepsilon ,\upvarepsilon } \right)$$

$$\phi^{^{\prime}} ,\phi_{0}^{^{\prime}} ,\phi_{1}^{^{\prime}} \sim{\text{normal}}\left( {0,\frac{1}{{\upvarepsilon ^{2} }}} \right) \quad\upvarepsilon = 0.1$$

$${\text{r}},{\text{r}}_{{\text{k}}} ,{\text{r}}_{{\text{k}}} \sim{\text{Normal}}\left( {0,1} \right)$$

$${\upnu }^{^{\prime}} ,{\text{v}}_{0}^{^{\prime}} ,{\text{v}}_{1}^{^{\prime}} \sim{\text{Normal}}\left( { - 2,1} \right)$$

### Analysis of the data

**Fig. 1:**
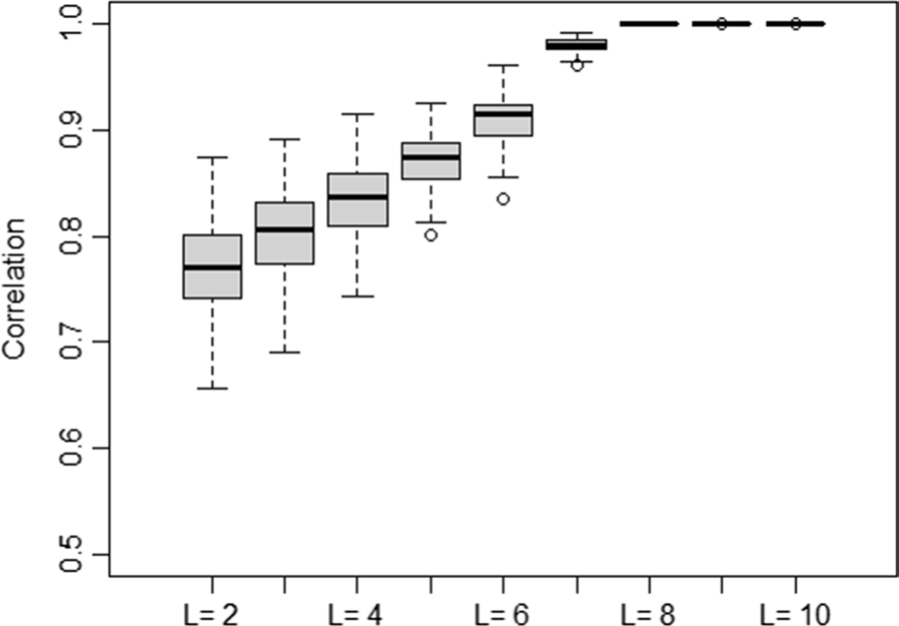
Exploratory plot for the cortical thickness data. Legendre resolution, L., provides a correlation between the original and rebuilt data. The correlations of the 53 subjects are shown in each boxplot

In the present study, the Chi-square test was used to check the homogeneity between groups in terms of gender distribution, and a one-way analysis of variance (ANOVA) was used to compare means of age across groups. A fully spectral Bayesian model was applied to the cortical thickness data related to the TLE (right and left) patients and healthy controls. The value of L = 7 was considered, which reduced the number of observations per subject from 68 in the spatial domain to 64 in the spectral domain. According to exploratory analysis (Fig. 1), terms beyond L = 7 did not appear to have any additional spatial signals.

The Markov chain Monte Carlo (MCMC) algorithm was run for 100,000 iterations so that the first 10,000 iterations were discarded as burn-in period, and the rest of them were thinned by 10 to eliminate autocorrelations. Considering the Matérn correlation structure and $J = 9$ basis functions, fitting the full model to the epilepsy data took about 2 min for every 1000 iterations.

Several settings were considered to find the best-fitted models, among others. The fit of the models varies depending on the residual correlation, fixed and random effects, and the number of basis functions $J$. A leave-one-out cross-validation (LOOCV) strategy was considered to compare different models because the sample size was small. Thus, the model was applied once for each subject, using all other subjects as a training set and using the selected subject as a single-item test set. The results of the mean squared error of the posterior predictive means were provided in Table 2 to compare different methods.

**Table 2:** The cortical thickness data underwent cross-validation

Residuals	FE	Nonstationary	*J*	MSE
Independent	Gaussian	No	4	4.85E−02
Independent	Gaussian	No	9	4.78E−02
Independent	Gaussian	Yes	4	4.15E−02
Independent	Gaussian	Yes	9	4.05E−02
Independent	Horseshoe	No	4	4.79E−02
Independent	Horseshoe	No	9	4.77E−02
Independent	Horseshoe	Yes	4	4.11E−02
Independent	Horseshoe	Yes	9	4.09E−02
Matérn	Gaussian	No	4	4.74E−02
Matérn	Gaussian	No	9	4.78E−02
Matérn	Gaussian	Yes	4	4.59E−02
Matérn	Gaussian	Yes	9	4.01E−02
Matérn	Horseshoe	No	4	4.83E−02
Matérn	Horseshoe	No	9	4.90E−02
Matérn	Horseshoe	Yes	4	4.16E−02
Matérn	Horseshoe	Yes	9	4.22E−02

The residual correlation of $\tilde{E}$ affects the strategies used to fit the data; the prior for the fixed effects (“FE”) $\tilde{B}$, the inclusion of the nonstationary subject random effects (“nonstationary”) $\tilde{Z}$, and the number of basis functions J. Methods are compared by the mean squared prediction error (MSE).

The Gaussian prior for fixed effects was sufficient for the data used in this study, so the Gaussian priors for fixed effects and covariance with J = 9 basis functions that include both the stationary Matérn component and the nonstationary component were used.

For the z-score maps, the threshold produced using the method of Sun et al. [39] to control the Bayesian false discovery rate at 0.01. In this multiple testing problem, the one-sided null and alternative hypotheses were $H_{0} :\beta_{K} \left( s \right) \ge 0$ and $H_{1} :\beta_{k} \left( s \right) < 0,$ respectively. The results were declared to be statistically significant while controlling the Bayesian false discovery rate at 0.01 using the method of Sun et al., if z <  − 0.984 for age, z <  − 0.390 for left TLE comparing to healthy controls, z <  − 0.049 for right TLE patients comparing to healthy controls, z <  − 0.530 for left TLE patients comparing to right TLE.

After identifying significant regions with the fully spectral Bayesian model, a multivariate analysis of covariance (MANCOVA) was conducted to compare the means of cortical thickness across groups adjusting for age and gender to assess if the mean of the cortical thickness of the identified regions has statistically significant differences across three groups. Also, we used artificial neural networks technique for classification of TLE patients and healthy controls to see the discrimination power of identified regions. In this regard, a commonly used multi-layer perceptron approach with three layers was considered, including an input layer (using the identified brain regions), a hidden layer and an output layer. In this model, the neurons applied a nonlinear activation function to calculate the outputs (here, a categorical output with TLE and healthy controls as categories). In this study, a sigmoid function (f(x) = 1/(1 + exp(− x))) was used as the activation function in the hidden layer (achieved a better accuracy among others) and a linear function was used in the output layer. A LOOCV strategy was used to optimize the network. A classical vertex-by-vertex linear regression was used to identify regions with reduced cortical thickness. The artificial neural networks classifiers were also constructed using the regions identified by the classical method. Comparisons were done based on the area under the ROC curve.

## Results

Fifty percent of the healthy controls, 58% of left TLE patients, and 72% of right TLE patients were male, where the chi-square test revealed that the distribution of gender was not statistically significant across groups (Chi-squared = 1.56; df = 2; *p* = 0.458). The average ($\pm {\text{ standard deviation}}$) age of the participants was $32.10{ } \pm { }8.47$, $28.28{ } \pm { }6.28,{\text{ and }}27.95 \pm 6.32$ for left TLE patients, right TLE patients, and healthy controls, respectively. The one-way ANOVA revealed that there were not statistically significant differences between groups in terms of age (F = 1.92; df1 = 2, df2 = 50; *p* = 0.157).

**Fig. 2:**
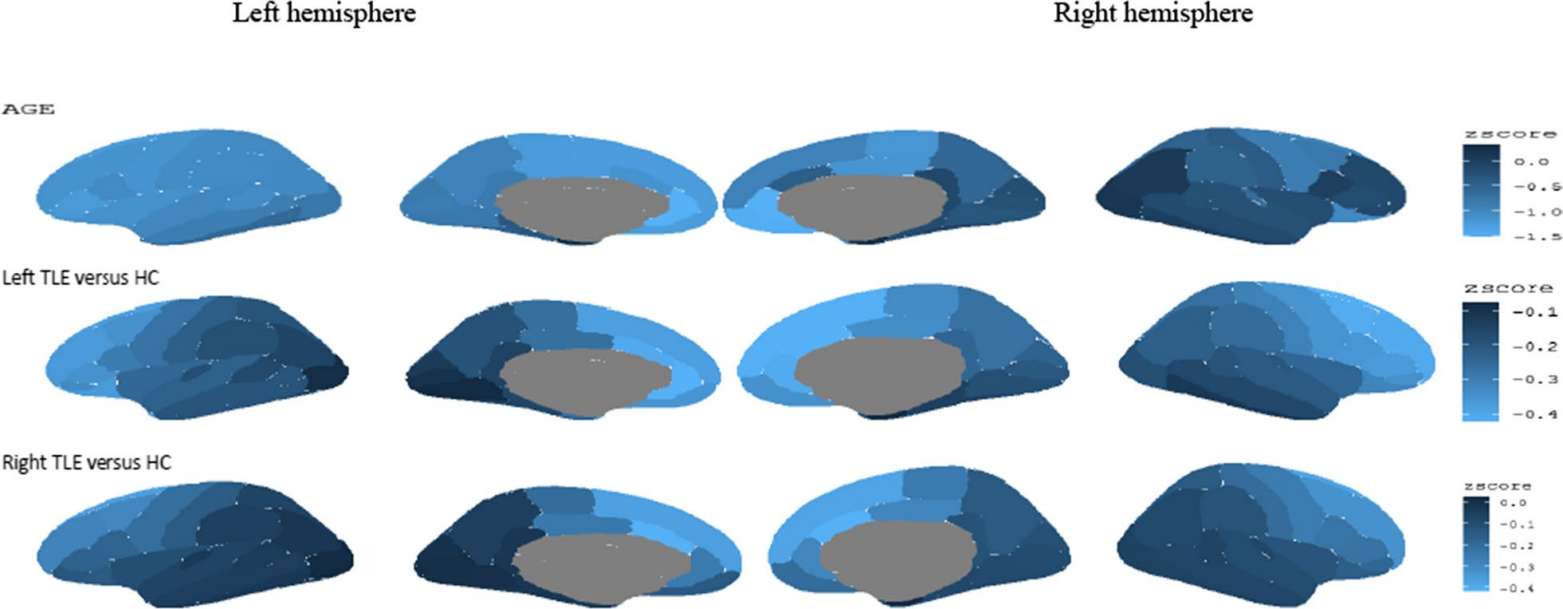
Posterior z-scores (i.e., the ratio of the posterior mean and standard deviation) for the fixed effects for cortical thickness data (the intercept effect is omitted). The medial wall, where the two hemispheres meet, is indicated in the center of the photographs in the middle two columns, although it does not include measures of cortical thickness. In the figure, the HC indicates the healthy controls

**Fig. 3:**
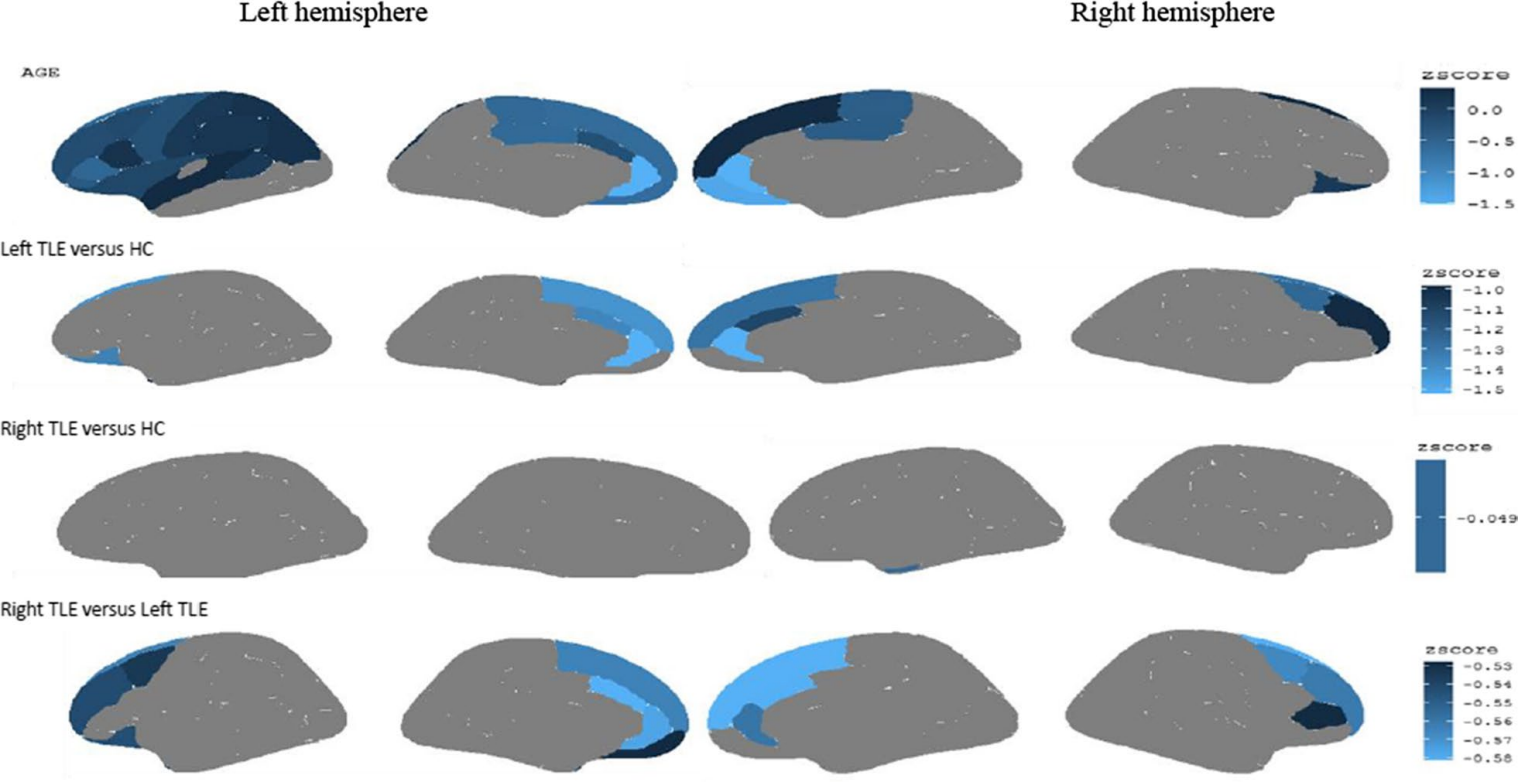
Regions of significant cortical thinning in patients with temporal lobe epilepsy according to the Bayesian false discovery rate. Values are posterior z-scores for the fixed effects for data of cortical thickness. Estimates were grayed out for the regions that were not statistically significant according to the Bayesian false discovery rate as clarified in the method section). In the figure, the HC indicates the healthy controls

The variables of age, gender of the participants, and the groups of participants (left TLE, right TLE and HC) were considered as the covariates in the spectral Bayesian model, and their local effects were evaluated. Posterior z-scores (i.e., the ratio of the posterior mean and standard deviation) of the fixed effects for cortical thickness data were plotted in Fig. 2. Regions of the cerebral cortex that are darker blue have larger (more positive) posterior Z-scores. Also, the lighter colors indicate the more negative Z-scores. The greater negative values indicate more atrophy in each region of the cerebral cortex. The first row in Fig. 2 shows the posterior z-scores of age, the second row in Fig. 2 shows the posterior z-scores of the left TLE, and the third row in Fig. 2 shows the posterior z-scores of the right TLE (the HC was considered as the baseline group). According to the threshold produced in “Statistical analysis” section (z <  − 0.984), age had strong associations in the anterior, specifically in the bilateral Paracentral gyrus, medial orbitofrontal gyrus, lateral orbitofrontal gyrus, and superior frontal gyrus. The first row in Fig. 3 depicts statistically significant regions.

### Cortical thinning in left TLE compared to controls

According to the threshold produced in “Statistical analysis” section (z <  − 0.39), the comparison of cortical thickness abnormalities between control and left TLE patient groups revealed clusters of cortical thinning over the left hemisphere, mainly on the cingulate lobe (caudal anterior cingulate, rostral anterior cingulate), the frontal lobe (superior frontal, lateral orbitofrontal, frontal pole) and the temporal lobe (temporal pole). In the right hemisphere, also, clusters of cortical thinning were observed on the cingulate lobe (caudal anterior cingulate, rostral anterior cingulate) and the frontal lobe (caudal middle frontal, rostral middle frontal, superior frontal). These significant regions are listed in Table 3, and the second row in Fig. 3 depicts the statistically significant regions.

**Table 3:** Cluster-based model of cortical thinning for left TLE patients vs. Healthy controls

Cluster lobe	Hemisphere	Z-score	Cortical thickness mean $\pm$ SD	
			HC	Left TLE
Caudal anterior cingulate	L	− 1.15	2.83 ± 0.23	2.73 ± 0.17
Lateral orbitofrontal	L	− 1.10	2.76 ± 0.12	2.72 ± 0.15
Rostral anterior cingulate	L	− 1.52	2.97 ± 0.19	2.84 ± 0.21
Superior frontal	L	− 1.26	2.77 ± 0.21	2.67 ± 0.19
Frontal pole	L	− 1.31	2.93 ± 0.31	2.84 ± 0.25
Temporal pole	L	− 0.54	3.76 ± 0.26	3.65 ± 0.49
Caudal anterior cingulate	R	− 0.82	2.68 ± 0.21	2.62 ± 0.31
Caudal middle frontal	R	− 0.95	2.55 ± 0.19	2.39 ± 0.09
Rostral anterior cingulate	R	− 1.22	2.96 ± 0.20	2.90 ± 0.22
Rostral middle frontal	R	− 0.70	2.45 ± 0.14	2.38 ± 0.14
Superior frontal	R	− 1.00	2.71 ± 0.18	2.56 ± 0.18

*HC* Healthy controls, *L* Left, *R* Right, *TLE* Temporal lobe epilepsy

### Cortical thinning in right TLE compared to controls

According to the threshold produced in “Statistical analysis” section (z <  − 0.049), the comparison of cortical thickness abnormalities between healthy controls and right TLE patient groups revealed clusters of cortical thinning over the right hemisphere on the temporal lobe (entorhinal). This significant region is listed in Table 4, and the third row in Fig. 3 depicts the statistically significant region.

**Table 4:** Cluster-based model of cortical thinning for right TLE patients vs. Healthy controls

Cluster lobe	Hemisphere	Z-score	Cortical thickness mean $\pm$ SD	
			HC	right TLE
Entorhinal	R	0.04	2.81 ± 0.22	2.71 ± 0.17

*HC* Healthy controls, *R* Right, *TLE* Temporal lobe epilepsy

### Cortical thinning in left TLE compared to right TLE

According to the threshold produced in “Statistical analysis” section (z <  − 0.53), the comparison of cortical thickness abnormalities between left TLE and right TLE patient groups revealed clusters of cortical thinning over the left hemisphere on the cingulate lobe (caudal anterior cingulate, rostral anterior cingulate) and frontal lobe (superior frontal, caudal middle frontal, lateral orbitofrontal, rostral middle frontal, frontal pole). The right hemisphere, also, showed clusters of cortical thinning on the cingulate lobe (caudal anterior cingulate, rostral anterior cingulate), and the frontal lobe (caudal middle frontal, rostral middle frontal, pars triangularis, superior frontal). These significant regions are listed in Table 5, and the fourth row in Fig. 3 depicts the statistically significant regions.

**Table 5:** Cluster-based model of cortical thinning for left TLE vs. right TLE patients

Cluster lobe	Hemisphere	Z-score	Cortical thickness mean $\pm$ SD	
			Right TLE	Left TLE
Caudal anterior cingulate	L	− 0.56	2.83 ± 0.28	2.73 ± 0.17
Caudal middle frontal	L	− 0.53	2.51 ± 0.14	2.39 ± 0.12
Lateral orbitofrontal	L	− 0.54	2.74 ± 0.12	2.72 ± 0.15
Medial orbitofrontal	L	− 0.52	2.52 ± 0.21	2.51 ± 0.15
Rostral anterior cingulate	L	− 0.56	2.89 ± 0.20	2.84 ± 0.21
Rostral middle frontal	L	− 0.54	2.46 ± 0.16	2.44 ± 0.21
Superior frontal	L	− 0.55	2.69 ± 0.19	2.67 ± 0.19
Frontal pole	L	− 0.53	2.59 ± 0.29	2.93 ± 0.31
Temporal pole	L	− 0.53	3.80 ± 0.38	3.65 ± 0.49
Caudal anterior cingulate	R	− 0.58	2.82 ± 0.28	2.62 ± 0.31
Caudal middle frontal	R	− 0.56	2.51 ± 0.14	2.39 ± 0.09
Pars triangularis	R	− 0.52	2.48 ± 0.16	2.44 ± 0.13
Rostral anterior cingulate	R	− 0.55	2.92 ± 0.20	2.90 ± 0.22
Rostral middle frontal	R	− 0.56	2.46 ± 0.16	2.38 ± 0.14
Superior frontal	R	− 0.58	2.68 ± 0.18	2.56 ± 0.18

*HC* Healthy controls, *L* Left, *R* Right, *TLE* Temporal lobe epilepsy

### Classification

**Fig. 4:**
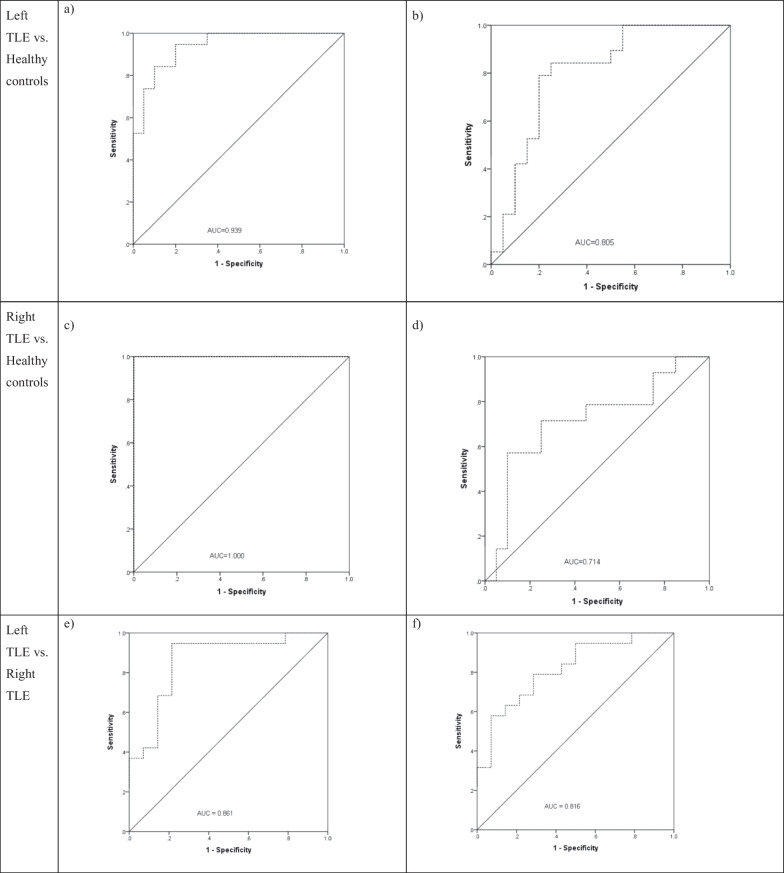
ROC curves for classification of TLE patients versus Healthy controls and left versus right TLE patients using Neural Networks based on identified brain regions in Table 3 and 4 (figures **a**, **c** and **e**) as well as those obtained by a vertex-by-vertex general linear model (figures **b**, **d**, **f**)

The identified regions with decreased cortical thickness were entered into a MANCOVA to compare the three groups. The results provided a significant difference between three groups (F = 122.799; df_1_ = 2, df_2_ = 48); *P* < 0.001) in terms of mean of the cortical thickness of the identified regions, confirming that the identified regions have different average of the cortical thicknesses across the three groups. Then, the artificial neural network-based classifiers were constructed using the identified brain regions with decreased cortical thicknesses as the inputs. Figure 4 shows the ROC curves related to the four neural networks. The right panel ((a) and (c)) illustrates the ROC curves related to the neural networks created by the identified brain regions with decreased cortical thickness using the fully Bayesian method and the left panel ((b) and (d)) illustrates the ROC curves related to the neural networks created by the identified brain regions based on a vertex-by-vertex linear regression (identified regions related to the left TLE patients versus healthy controls included caudal middle frontal, cuneus and supra marginal on the left and caudal middle frontal, supra marginal superior parietal on the right hemisphere of the brain; and identified regions related to the right TLE patients versus healthy controls included superior frontal, supra marginal, medial orbitofrontal and supra marginal). According to the results, the former provided better prediction accuracies than the later, so that for classification of the right TLE versus healthy controls, the AUC in classification of patients using neural network was 1.000, while it was 0.714 for the neural network created based on the regions using the vertex-by-vertex regression. This was also the case for classification of the left TLE versus healthy controls, where the AUC based on the regions obtained by the fully Bayesian model was 0.936 and it was 0.805 for the vertex-by-vertex regression. Moreover, the AUC in classification of left versus right TLE patients using obtained regions in the fully Bayesian method was 0.861, while it was 0.816 based on identified regions from the classical vertex-by-vertex regression.

## Discussions

This study applied a spatial-spectral model for analyzing MRI data in patients with TLE to identify regions with changes in cortical thickness compared to healthy controls. The used model utilizes the Bayesian framework that takes advantage of the spherical harmonics transformation to deal with the spherical nature of the datasets. Here, the stationary Mat'ern component provided a better fit to the data compared to the independent structure. The used spatial model identified regions of the cortex that differed by the TLE status. Due to the complex structure of MRI data, utilizing statistical methods that account for characteristics like nonstationary spatial correlations between regions and local covariate effects is of great importance; a property that are often neglected in modeling these types of data.

Recently, the assessment of brain atrophy and brain structural changes using neuroimaging methods have been widely used to track the inception/progression of neurodegenerative diseases. In TLE, epileptogenicity can produce cortical thinning in the temporal or extratemporal lobes neocortical regions [40–43]. Also, in patients with TLE, it has been shown that the structural abnormalities extend beyond the temporal lobe [44], and different patterns of neocortical atrophy or cortical thinning have been reported. Studies have demonstrated that TLE is associated with a high degree of brain atrophy that extends further into the limbic system and temporal lobe regions, including the amygdala and thalamus volumes [45] as well as the anteromedial regions, including the entorhinal cortex, temporal pole, and parahippocampal gyrus [46, 47] and regions that are functionally and anatomically connected to the hippocampus [8, 48]. Moreover, both temporal and extra-temporal regions have been reported to be affected by TLE. The hippocampal atrophy (ipsilateral to the epileptogenic) and bilateral hippocampal structural changes have been reported by several studies in patients with left [18, 43, 49] and right TLE [19, 50].

Our findings about the cortical thickness revealed that in the left TLE patients, the cortical region of caudal anterior cingulate had a significant thinning compared to the control group, bilaterally. Ogren et al. [51] have investigated regional cortical thickness changes accompanying generalized tonic–clonic seizures. However, they did not find a significant difference in this region between patients (n = 53) and healthy controls (n = 530). This might be due to the differences in recruited samples in terms of considering older patients on average (age average: 37 ± 12.6) in their study compared to the present study (age average: 29.53 ± 7.29). The observed disagreement can also be the result of using different approaches to analyze the data (i.e. independent samples t-test in their study by ignoring spatial correlations between regions versus model-based in our study). Changes in caudal anterior cingulate (cortical thickening) have been reported by studies in psychosis of epilepsy compared to epilepsy-without psychosis patients [52]. The observed inconsistency highlights the need for more investigations using larger sample sizes and advanced statistical models through well-designed studies.

Moreover, our findings revealed a significant reduction (i.e., thinning) in the cortical thickness in the ipsilateral lateral orbitofrontal cortex in the left TLE patients compared to the control group. This finding was also in agreement with the findings of other studies [48, 53]. The bilateral cortex thinning of rostral anterior cingulate region was observed in this study which was in agreement with previous studies [54]. The bilateral cortex thinning was observed in the superior frontal region as well in the left TLE patients compared to the control group. Other studies have reported a contralateral side thinning [20] and some others have reported the cortex thinning in the ipsilateral side in patients with TLE [55] and focal epilepsy [42]. We observed that in addition to temporal regions, the frontal regions are also involved in cortical thinning in the left TLE patients compared to healthy controls. For example, the cortex thinning of the frontal pole and the temporal pole was observed on an ipsilateral side. Moreover, the cortex thinning of cortical regions of the caudal middle frontal and the rostral middle frontal was observed in a contralateral side in the left TLE patients compared to healthy controls. These findings was consistent with the results of other studies [43].

For the right TLE patients, the only cortical region with significant thinning was the entorhinal region (compared to the healthy controls) on the ipsilateral side. This finding was in agreement with the results of several studies [8, 56–58]. Bernasconi et al. showed that there was a bilateral reduction in the volume of the entorhinal cortex in unilateral TLE patients compared with the healthy controls [59]. Our findings indicated that more regions had changes in the left TLE patients than the right TLE patients. This finding was in concordance with other studies [20, 30, 43, 44, 58, 60–62]. Classifications using the artificial neural networks technique based on the identified regions in this study provided a greater prediction accuracies (AUCs were 1.000 for right TLE patients versus healthy controls and 0.936 for left TLE patients versus healthy controls) compared to the regions identified by the traditional statistical models (AUCs were 0.714 for right TLE patients versus healthy controls and 0.805 for left TLE patients versus healthy controls) indicating the potential usefulness of the utilized fully Bayesian model in identifying regions with thinning in the cortical thickness.

We, also, considered the local effect of age on different regions. This allowed us to have a region-by-region effect of age which varies over the regions considering the correlation between the regions with complex structures [35]. Our findings suggested that age had strong effects on the bilateral medial orbitofrontal gyrus, lateral orbitofrontal gyrus, and superior frontal gyrus regions. This finding points to a remarkable amount of cortical plasticity in primary motor and visual regions which is consistent with other studies [63].

In the present study, we considered a node-based cortical thickness analysis. A node-based approach has advantages for the brain structure and function analysis over the voxel-based approaches, for example: reducing the effect of noise on the pixels of MR images and also mis-segmentation of gray and white matter boundaries. In addition, since each brain anatomical region, which includes several voxels, has a specific function, using the node-based method is preferred to the voxel-based analysis because of identifying the regions affected by TLE.

In this study, a spatial-spectral model for imaging datasets was used to analyze the cortical thickness in temporal lobe epilepsy by MRI data. This model deals with nonstationary spatial covariance structure as well as local covariate effects. Also, the fully Bayesian model uses the spherical harmonics transformation. This feature makes the application of the model feasible for large datasets while the spherical nature of the data is maintained. Reich et al. showed that taking into account the residual spatial correlation is essential for efficient estimation and valid inference. They also demonstrated through simulation studies that the model with stationary covariance and independent residuals often gives high MSE and low coverage, and inclusion of nonstationarity as well as horseshoe priors improves performance of the model [35]. While the used Bayesian approach enjoys the above advantages, there is a need for a strong background in the Bayes and spatial data analysis for researchers to use it. Also, the model is computationally expensive compared to the classic likelihood-based analysis and there is a need to use parallel computing to reduce the analysis time. However, regarding the small sample size (which is usually the case for MRI research), the Bayesian framework provides more reliable results compared with the classical methods. Also, regarding a large number of random effects, calculating the integrals over them would be very expensive, which makes the Bayesian framework an interesting approach for obtaining estimates [64, 65].

## Limitations

This study had some limitations in the analysis of cortical thickness data. The data on the duration of the disease, severity of the disease and the number of seizures were not available. This would confound the results of this study. Also, the sample size in this study was small, so we were unable to find a link between gray matter atrophy and seizure frequency. Besides the limitations of this study, we used a powerful statistical model suitable for high dimension data to analyze MRI data that accounts for the spatial correlation between brain regions.

## Conclusions

We used a fully Bayesian spectral method to analyze gray matter anomalies in the cortex for temporal lobe epilepsy patients in this study. According to our findings, the thickness of cortical gray matter is influenced by temporal lobe epilepsy, and left TLE patients had a higher chance of cortical thickness anomalies compared to the right TLE patients. More investigations using larger sample sizes are crucial to validate the results of this study.

## Data Availability

The datasets generated during and analyzed during the current study are not publicly available due to the legacy Iranian National Brain Mapping Laboratory (NBML) restrictions on public sharing data, but are available from the corresponding author upon reasonable request.

## Abbreviations

ANOVA: Analysis of variance
HC: Healthy controls
L: Left
NBNL: National Brain Mapping Lab
R: Right
SD: Standard deviation
TLE: Temporal lobe epilepsy
MSE: Mean square error
MRI: Magnetic resonance imaging
MANCOVA: Multivariate analysis of covariance
LOOCV: Leave-one-out cross-validation
MCMC: Markov chain Monte Carlo
